# Partitioning two components of BOLD activation suppression in flanker effects

**DOI:** 10.3389/fnins.2014.00149

**Published:** 2014-07-08

**Authors:** Chien-Chung Chen

**Affiliations:** ^1^Department of Psychology, National Taiwan UniversityTaipei, Taiwan; ^2^Neurobiology and Cognitive Science Center, National Taiwan UniversityTaipei, Taiwan

**Keywords:** collinearity, m-sequence, lateral interaction, flanker effect, spatial configuration

## Abstract

The presence of a visual stimulus not only increases the blood oxygenation level dependent (BOLD) activation in its retinotopic regions in the visual cortex but also suppresses the activation of the nearby regions. Here we investigated whether there are multiple components for such lateral effects by using the m-sequence paradigm to measure the stimulus spatial configuration specific BOLD activation. The central target (2 cyc/deg grating) was centered on a fixation point while the flanking stimulus was placed 2° away and was located on axes that were either collinear or orthogonal to the target's orientation. Three types of flankers were used: gratings whose orientation was the same as the central stimulus, gratings which were orthogonal to the stimulus, and random dots. The onset and offset of each stimulus were determined by shifted copies of an 8-bit long m-sequence. The duration of each state of the sequence was 2 s or 1TR. The first order activation, computed as the waveform recorded following on-states minus that recorded after off-states, determined the retinotopic regions for each stimulus. We then computed BOLD activation waveforms for the target under various flanker conditions. All flankers reduced the activation to the target. The suppressive effect was largest following the presence of the iso-orientation collinear flankers. Our result suggests two types of BOLD signal suppression: general suppression, which occurs whenever a flanker is presented and is insensitive to the spatial configuration of the stimuli, and spatial configuration dependent suppression, which may be related to the collinear flanker effect.

## Introduction

The visual response to a stimulus can be modulated by another stimulus. For instance, in the Ebbinghaus effect, a target circle surrounded by large circles appears to be smaller than the same target surrounded by small circles; in simultaneous contrast (Wallach, 1948; Gilchrist, 2006), a patch of gray on a dark background appears brighter than the same patch on a bright background; and, in particular, in the flanker effect (Polat and Sagi, 1993, 1994; Chen and Tyler, 2001, 2008), the visibility of a low contrast periodic pattern (target) increases when it is flanked by collinear and iso-oriented patterns (flankers). Such lateral modulation of visual performance may have a neurophysiological basis. Whereas a visual cortical neuron only responds to visual stimuli projected onto its receptive field (Hubel and Wiesel, 1962; DeAngelis et al., 1993), this response can be modulated by the presence of other visual stimuli presented outside its classical receptive field (Blakemore and Tobin, 1972; Nelson and Frost, 1985; Knierim and Van Essen, 1992; Sillito et al., 1995; Polat et al., 1998; Sengpiel et al., 1998; Kapadia et al., 1999, 2000; Chen et al., 2001; Freeman et al., 2001; Angelucci et al., 2002; Cavanaugh et al., 2002).

In psychophysical or electrophysiological experiments, the lateral effect can be measured by comparing the visual performance or cell response to a visual target in the presence of a spatial context with those without a context. This approach, however, may not be directly applicable to an fMRI study. The blood oxygenation level dependent (BOLD) activation in the early visual cortex does show a retinotopic property, i.e., that the activation of a particular voxel corresponds to the presence of visual stimuli at a certain location (Engel et al., 1997; Tootell et al., 1998). Thus, at first glance, it might be possible simply to measure the context effect by comparing the activation of a set of voxels to a target projected to their corresponding retinotopic locations on the display, with and without the presence of a context outside their corresponding retinotopic locations. Indeed, there were fMRI studies did just that (Zenger-Landolt and Heeger, 2003; Tajima et al., 2010; Wade and Rowland, 2010). However, the result from such experimental paradigm may not reflect the true neural mechanisms for context effect. For instance, it is known that the presence of a visual stimulus not only produces an increment of BOLD activation in the corresponding retinotopic regions for that stimulus, there is also a sustained reduction in BOLD activation in the neighboring brain regions (Logothetis, 2002; Shmuel et al., 2002, 2006; Smith et al., 2004; Chen et al., 2005). Such negative BOLD may not have a neurophysiological origin but, as discussed by Shulman et al. (1997) and Shmuel et al. (2002), it may be caused by “blood steal,” i.e., the activation to the visual stimuli draws fresh blood to the corresponding retinotopic region, thus reducing it in the neighboring regions. The reduction of fresh blood, in turn, causes a reduction in BOLD activation. Hence, there is a possibility that the effect of a context stimulus on the activation of a target brain region is simply caused by the regions responsive to that context stimulus drawing blood away. That is, the measurement of the context in the target region may be contaminated by a hemodynamic cause and thus cannot reflect the nature of the lateral interactions between neural mechanisms.

The possibility of the involvement of a hemodynamic factor in the BOLD activation illustrates the risk of studying context effect. Even without the hemodynamic factor, the presentation of the context stimulus may cause an overall change in the neural activity due to, say, an increment in the stimulus size. Thus, the experimental result may tag a neural mechanism that is unrelated to the context effect in perception. To avoid such risk, the better strategy is to compare activation to the stimuli that are known to cause a difference in perception.

The flanker effect is strongly configuration dependent. At the behavioral level, the detection threshold for a Gabor target is reduced by the presence of Gabor flankers only if the flanker has the same orientation (Polat and Sagi, 1993; Chen and Tyler, 2002) and is placed on the collinear axis of the target orientation (Polat and Sagi, 1993, 1994; Solomon and Morgan, 2000; Chen and Tyler, 2008). A flanker with an orthogonal orientation or which is placed away from the collinear axis has little, if any, effect on target detection. Electrophysiological evidence also shows that the response of a visual cortical neuron is best modulated if the context has an orientation similar to the preferred orientation of the cell (Blakemore and Tobin, 1972; Nelson and Frost, 1985) and is placed on the collinear axis (Kapadia et al., 2000).

Here, we exploited the configuration dependency of the flanker effect. We tested the BOLD activation of the brain region responsive to a central target in the presence of flankers with different orientations and locations. A BOLD activation caused by the visual context effect should show a dependency on the spatial configuration. That is, a flanker which has the same orientation and is placed on the collinear axis should produce the largest change in BOLD activation from that to the target alone. On the other hand, a lateral effect that is not related to the visual context effect, such as those with hemodynamic origins or an overall change in neural activity, should be indifferent to the spatial configuration of the stimuli.

In our experiment, there could be more than one stimulus component on the display. To separate the effect of different stimulus components, we used an M-sequence technique (Sutter, 2001; Buracas and Boynton, 2002) to control the experimental sequence. An M-sequence is a temporal binary (e.g., 0/1) random sequence that determines the state of a stimulus; in our experiment, the onset and the offset of the stimulus components. This type of binary sequence is generated in such a way as to consist of the same number of zero and one events and all possible combinations of zero and one events within a pre-designated length. That is, there is no bias on any states of the stimulus. Furthermore, an M-sequence also has the property that any temporal shift of the sequence is always orthogonal to the original sequence. Thus, one can assign each stimulus component to an M-sequence, which is a shift-copy of the M-sequences for other stimulus components. In this way, the occurrences of any stimulus component combinations, such as target alone, flanker 1 alone, target+flanker1, etc., are the same and therefore there is no bias toward any stimulus combination. In addition, since all M-sequences used in the experiments are orthogonal to each other, one can extract the effect of one stimulus component without being contaminated by the effect of the other components. With these properties, we were able to have multiple flankers in one fast event-related run and thus keep our experiment to a reasonable length.

## Methods

### Participants

Eight healthy volunteers between early 20 to early 40 years old participated in this study. One participant was the author of this paper while the others were naïve to the purpose of the experiment and were compensated financially for the hours of the experiment. Informed consent was obtained from each participant before scanning. The experiment was approved by the IRB of the National Taiwan University Hospital.

### Equipment and data acquisition

All stimuli were delivered with MR-compatible goggles (Resonance Technology, USA) mounted on the head of the participants. The resolution of the goggles was 800 × 600 with a dot size of 0.096° visual angle. The frame rate was 60 Hz. All the stimuli were generated on a PC compatible computer with the Psychophysics toolbox (Brainard, 1997) under the MATLAB (The Mathworks, Matick, MA, USA) environment. The visual acuity of the participants was corrected to normal by a set of convex lenses mounted on the goggles, in front of the display.

The magnetic resonance images were collected on a Bruker 30/90 Medspec 3T scanner (Bruker Medical, Ettlingen, Germany) with a cylindrical head coil. The functional images (T2^*^-weighted BOLD) were acquired with an Echo-planar imaging sequence (Stehling et al., 1991) with *TR* = 2000 ms, *TE* = 33 ms, flip angle = 90°, and voxel resolution = 3 × 3 × 3 mm. The images were collected in 20 transverse planes parallel to the AC-PC (anterior commissure-posterior commissure) line with a 19.2 cm FOV and an image matrix of 64 × 64. A set of anatomical images (T1-weighted, 256 × 256) was acquired in identical planes.

For the functional data, before statistical analysis, we first used SPM8 (http://www.fil.ion.ucl.ac.uk/spm/software/spm8/) software to correct for the timing difference between slices in a volume, and realigned the images acquired at different time points to remove head motion artifacts. The realigned images, as well as the anatomic images, were then normalized to a standard template with SPM8. The normalized images were fed to the mrVista software (Wandell et al., 2000) for co-registration and visualization after statistical analysis.

### Stimulus

**Figure 1:**
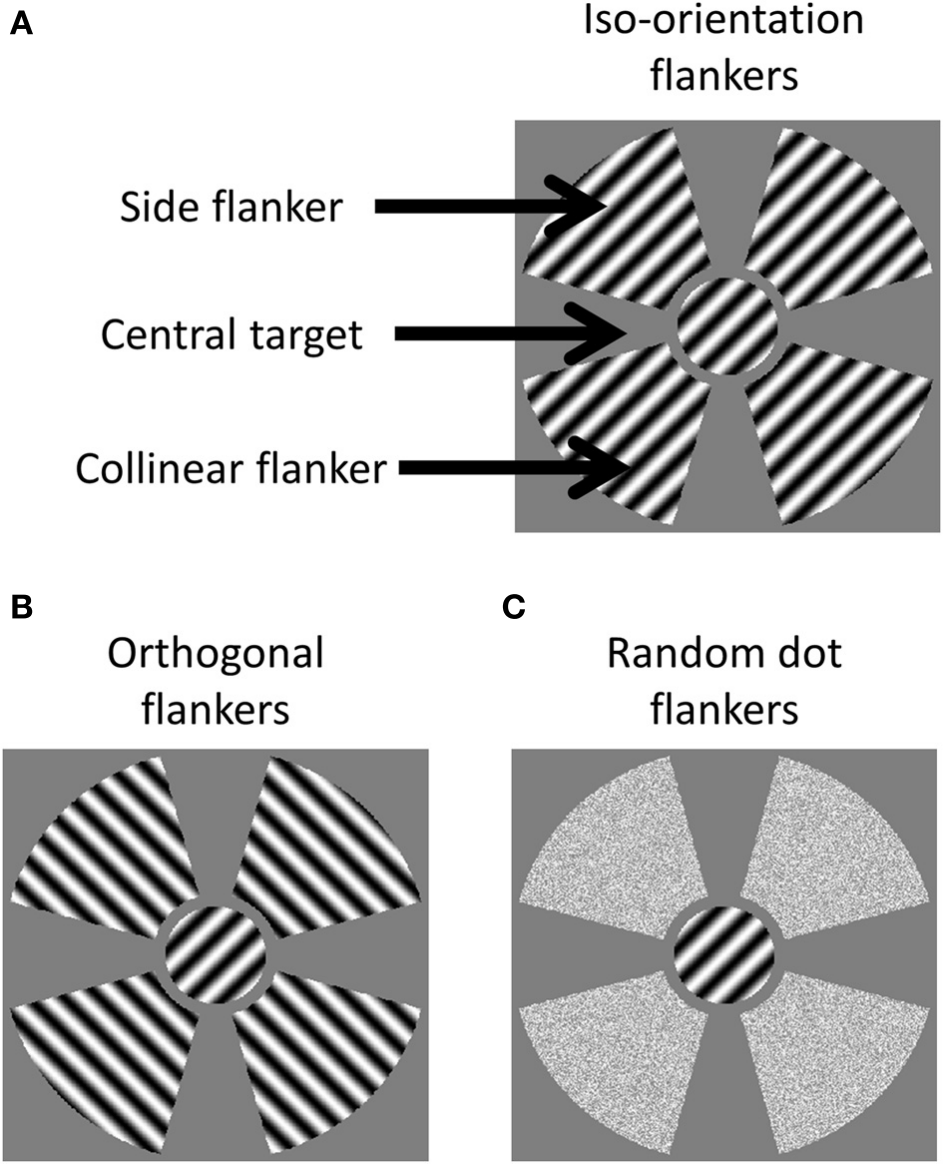
**cIllustration of stimuli in the three test conditions. (A)** The Iso-orientation condition with three image components labeled; **(B)** the orthogonal condition; and **(C)** the random dot condition.

As shown in Figure 1, there were three components in a stimulus. The first component was the central stimulus, or the target, which was a sinusoid grating with a 45° orientation, presented through a circular aperture with a 2° radius. The second component was a collinear flanker located on the two ends of the target along the axis that passed through the center and was parallel to the orientation of the target. The third component was a side flanker located on the axis orthogonal to the orientation of the target. The flankers were either a sinusoid grating or random dots presented through a fan aperture. The aperture in each quadrant extended from 2.5 to 6° visual angle from the center of the display in radius and spanned 70° in azimuth.

There were three types of stimulus. In the iso-orientation condition, the flankers contained sinusoidal gratings at a 45° orientation. In the orthogonal condition, the flankers contained sinusoidal gratings at a 135° orientation. The gratings had a spatial frequency of 4 cyc/deg and a contrast of 95%. In the random dot condition, the flankers were random dots whose luminance was drawn from a uniform distribution that had the same range and mean as the luminance distribution of the pattern stimuli. All stimulus components were presented on a gray background of mean luminance.

### Procedure

We used a fast event-related design. The stimulus was updated every 2 s (1TR). The sequence of the presentation of image components was determined by m-sequences. The method of generating m-sequences has been discussed by Sutter (2001). We used 8-bit m-sequences for the experiment. The duration of each state was 2 s. We used three shift-copies of the same sequence in each run. The original sequence controlled the onset and offset of the central target. The second sequence, which was constructed by shifting the first sequence by 64 states, controlled the collinear flanker. The third sequence, which was constructed by shifting the first sequence by 128 states, controlled the side flanker. The state value 1 meant that the image component was presented, while the state value 0 meant that it was not presented. In each state, the stimulus was presented for 1 s, followed by a 1 s blank in which only the gray background of mean luminance and the fixation point were shown on the display. All stimulus components, when presented, counter-phase flickered (that is, the luminance of each pixel alternating between positive and negative polarity about the mean luminance) at 4 Hz.

A circular fixation point (0.26° diameter) was placed at the center of the display throughout the experiment. At the beginning of each state, there was a 1/10 chance that the color of the fixation would change from red to green or *vice versa*. The observer was to press a button to indicate the change in fixation color. All observers achieved at least 80% accuracy in this fixation task.

For each participant, there were three functional runs, one for each of the iso-orientation, orthogonal orientation, and random dot conditions respectively. Each run started with a 6 s (3TR) blank period followed by 256 (2^8^) m-sequence states (512 s). The data from the first 6 s was not included in data analysis to avoid the start transient. The order of the three functional runs was randomized for each observer.

## Result

### The first order activation and ROI selection

**Figure 2:**
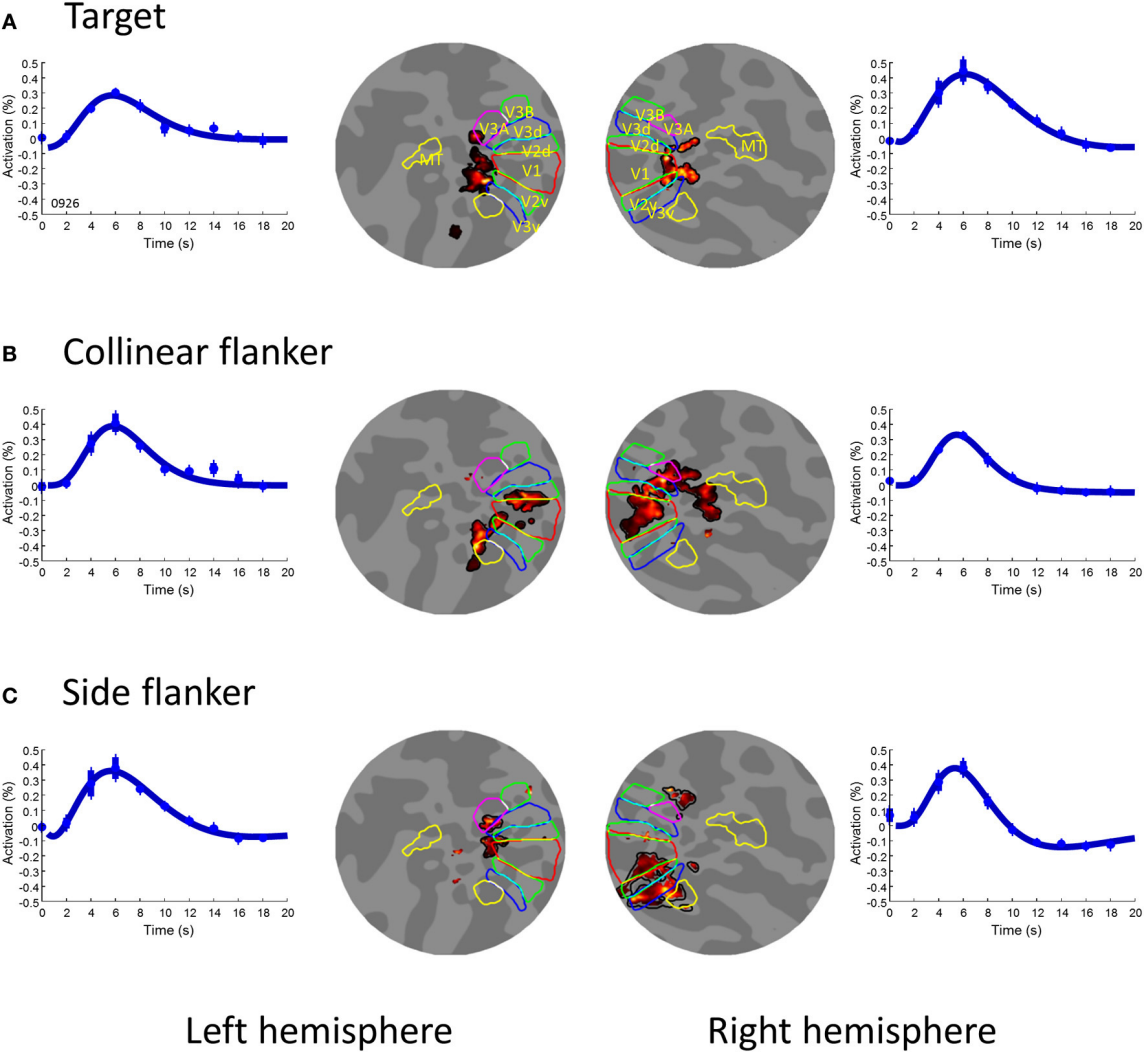
**The first order activation for the three image components. (A)** the target, **(B)** the collinear flanker, and **(C)** the side flanker. In each row, the two inner insets illustrated voxels (pseudo-colored area) showing significant [*t*_(253)_ > 4.72] activation to the respective image component on a flat map in the left and right hemispheres respectively. The colored contours denote visual areas as labeled in Panel **(A)**. The outer insets of each row are the waveform of activation averaged across significant voxels in the left and right hemispheres respectively. The smooth curve is the fit of a difference-of-gamma function. The error bars denote one standard error of measurement.

Figure 2 shows the first order activation for each stimulus component on flat maps for one observer. The flat maps here had their center near the occipital poles and extended 80 *mm* in radius around that point. The areas delineated by colored borders are the first-tier retinotopic areas (V1–3), identified with a rotating wedge for that observer acquired for a previous study (Chen et al., 2007).

The first order activation here is the BOLD activation to the presence of a stimulus component. The first order activation from an m-sequence can be extracted with a linear regression method (Buracas and Boynton, 2002). We first convolved the sequence for each image component with a difference-of-gamma (DOG) hemodynamic response function,

(1) $$\begin{array}{l} g\left(t\right)=w_{1}\times [{\left(t_{1}/\alpha_{1}\right)}^{\beta_{1}}\times e^{\left(-t_{1}/\alpha_{1}\right)}\\ \;-w_{2}\times {\left(t_{2}/\alpha_{1}\right)}^{\beta_{2}}\times e^{\left(-t_{2}/\alpha_{1}\right)}] \end{array}$$

where t is time in seconds, *t*_1_ = *t* − 6 and *t*_2_ = *t* − 12. The values of the parameters, given by Chen and Tyler (2008) are α_1_ = 5.4, α_2_ = 10.8, β_1_ = 6, β_2_ = 12, *w*_1_ = 1 and *w*_2_ = 0.35. Those parameters were shown (Chen and Tyler, 2008) to provide a good fit to the hemodynamic response function following a 1 s sensory stimulation measured by Glover (1999). The convolved sequences, along with a unity vector, were used as regressors. In this way, we were able to acquire the base line activation (the regression coefficient to the unity vector) and the activation amplitude to the presence of each of the three image components for each voxel. The activation of a voxel to an image component was considered significant if the t-statistics of the regression coefficient for the corresponding sequence reached 4.72. This criterion was equivalent to a two-way α-level about 10^−6^ for each individual voxel and Bonferroni corrected α-level 0.01, based on the number of gray matter voxels.

The central target produced activation in the foveal confluence region (Figure 2A). The two flankers, on the other hand, produced activation in the peripheral region (Figures 2B,C). The regions for the central target activation were used as areas of interest (ROIs) for the subsequent analysis. These ROIs respond little to the flankers alone. As shown in Figure 2 the areas activated by the flankers (Figures 2B,C) had no overlap with these ROIs. The amplitude of the BOLD activation in these ROIs to flankers never reached a statistically significant level (α-level 0.01). Hence, our result in the foveal ROIS cannot be explained by an intrusion from the flankers. Notice that, since it is difficult, if not impossible, to separate the foveal responses in different early visual areas, we opted to treat all voxels activated by the target in the early visual cortex in each hemisphere as one ROI.

### Lateral effects on BOLD activation

**Figure 3:**
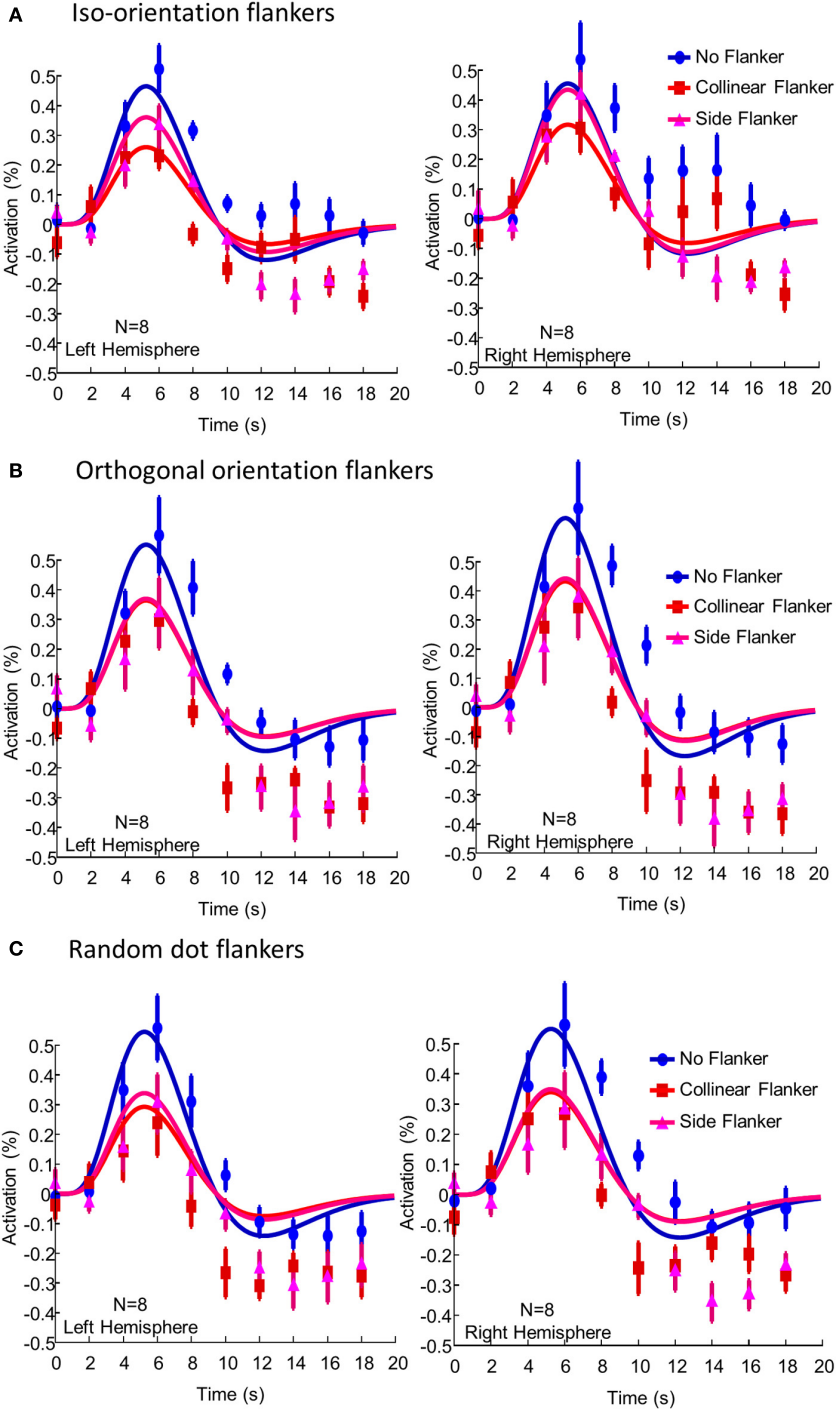
**The BOLD activation produced by the presence of the target in (A) the iso-orienation flanker condition, (B) the orthogonal flanker condition, and (C) the random dot flanker condition averaged across participants and voxels**. The left and right columns show activations in the left and right hemisphere ROIs respectively. In each panel, blue symbols and curves denote the BOLD activation following the stimulus events in which only the target was presented; red symbols and curves, the target and the collinear flanker; and magenta symbols and curves, the target and the side flankers. The smooth curves are fits of the difference-of-gamma function Equation (1). The error bars denote one standard error of individual difference.

Figure 3 showed BOLD activation produced by the presence of the target in the various flanker conditions in the left and right hemisphere ROIs. In each panel, blue symbols and curves denote the BOLD activation following the stimulus events in which only the target was presented; red symbols and curves, the target and the collinear flanker; and magenta symbols and curves, the target and the side flankers. The smooth curves are fits of the DOG function Equation (1) with amplitude w_1_ as the free parameter. The BOLD activation for each voxel was time locked average following a specific event. The activation was then averaged across all voxels in an ROI, before being averaged across participants. The error bars denote the standard error of individual difference.

Without flankers, the BOLD activation to the central target in the ROIs showed a typical biphasic shape and peaked at 6 s after stimulus onset. The presence of the flankers reduced the amplitude of BOLD activation to the target. In the iso-orientation condition, the presence of the flankers reduced the BOLD activation. The peak activation, on average, dropped 34% [*t*_(7)_ = 3.07, *p* = 0.009] and 27% [*t*_(7)_ = 3.21, *p* = 0.007] in the left and right hemisphere respectively. The activation with the collinear flankers was only half of that without flankers [*t*_(7)_ = 4.02, *p* = 0.003 for the left and *t*_(7)_ = 4.39, *p* = 0.002 for the right hemisphere]. Thus, while the presence of either flanker reduced the peak activation, the effect was greater in the collinear flanker condition than in the side flanker condition. The difference between the collinear and the side flanker was significant in both the left [*t*_(7)_ = 2.87, *p* = 0.01] and in the right hemisphere [*t*_(7)_ = 1.98, *p* = 0.04]. Notice that, the flankers also reduced BOLD activation in the undershoot region of the waveform. However, there was no systematic difference between the side and the collinear flankers in this.

In the orthogonal orientation condition (Figure 3B), the presence of either collinear or side flankers reduced the BOLD activation to the target. However, there was little, if any, difference in activation amplitude between the two flanker conditions. The result for the random condition (Figure 3C) was similar to that of the orthogonal orientation condition. That is, the presence of the flankers reduced the BOLD activation to the target by a similar amount regardless the location of the flankers.

**Figure 4:**
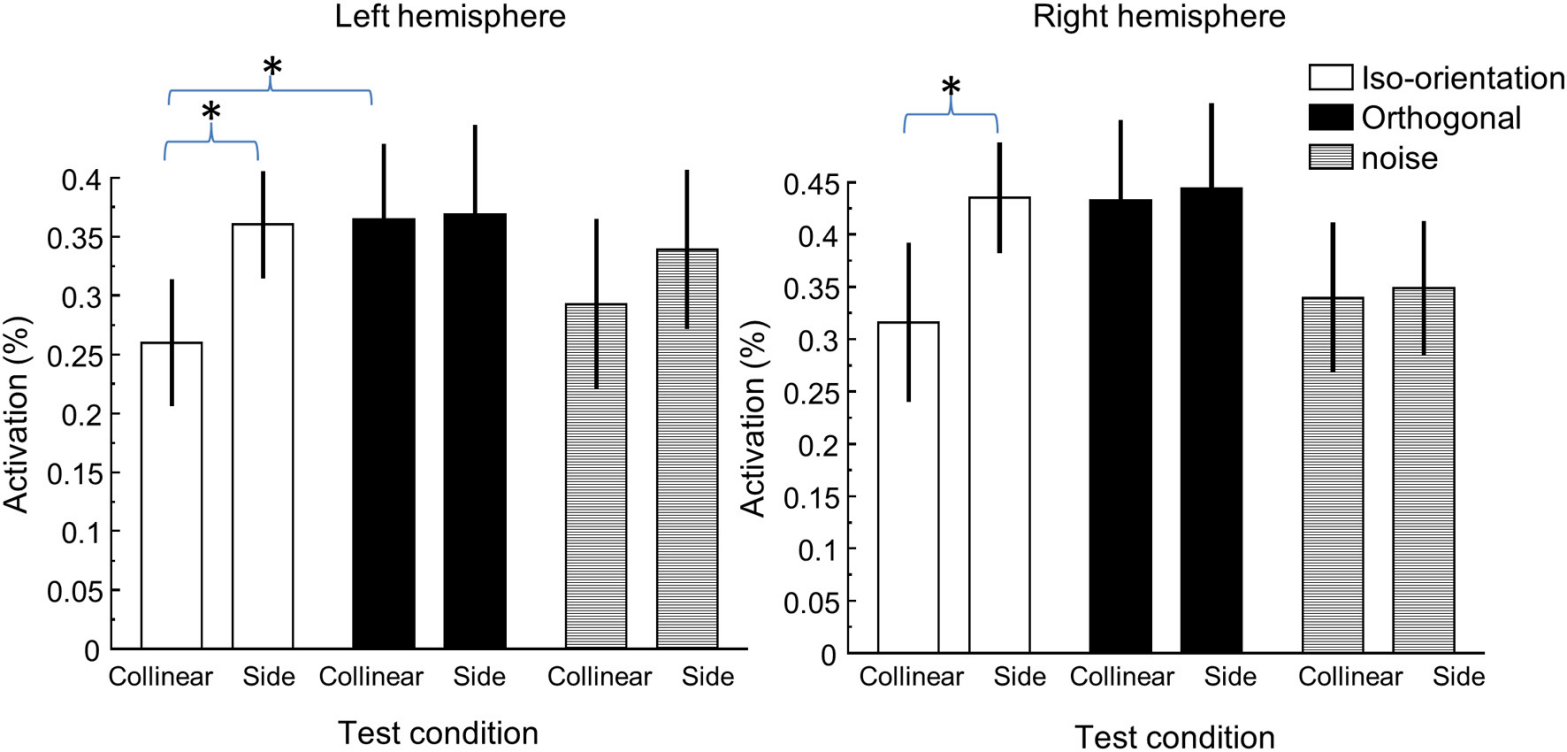
**The peak activation under various flanker conditions**. The left panel shows the activation in the left hemisphere ROI and the right panel shows the activation in the right hemisphere ROI. ^*^Denotes statistically significant difference at 0.05 level.

To summarize our result, Figure 4 shows the peak activation in all flanker conditions. As shown above, the flanker location effect, or the activation difference produced by the collinear and side flankers, was only significant in the iso-orientation condition. There was little, if any, difference in activation amplitude between the two flanker conditions in either orthogonal or noise conditions. The orientation effect, or the activation difference produced by the iso-orientation and orthogonal flankers was pronounced in collinear location. The difference was statistically significant in the left hemisphere [*t*_(7)_ = 2.01, *p* = 0.04] but not beyond the limitation of noise [*t*_(7)_ = 1.58, *p* = 0.08] in the right hemisphere. There was no orientation effect at the side location.

## Discussion

Despite a very short time interval between events (2 s), we were able to obtain a reliable measurement of BOLD activation to stimulus components (Figure 2) and various combinations of them (Figure 3) with m-sequences. Hence, the m-sequences technique is indeed a useful and efficient tool to measure brain activity to multiple visual inputs with fMRI.

In this study, we showed that BOLD activation to a target in the early visual cortical regions was suppressed by flankers presented outside the corresponding retinotopic locations of those regions. Such suppression occurred regardless of the orientation (iso-orientation, orthogonal orientation), composition (grating or random dot) or location (collinear or side) of the flankers. The suppression effect was greatest when the iso-orientation flankers were presented at the collinear location. Other than the iso-orientation collinear flankers, the suppression effect from all other flankers was similar. Hence, there seem to be at least two types of lateral suppression in the early visual cortex: one is a general suppression that occurs whenever a stimulus component is presented and the other is a spatial configuration specific suppression that occurs only when the iso-orientation collinear flankers are presented.

The configuration specific effect is consistent with the well-known collinear lateral interaction phenomenon, that is, that the visibility of a target periodic pattern can be altered by the presence of an iso-orientation flanker whose stripes are collinear with those of the target (Polat and Sagi, 1993, 1994; Zenger and Sagi, 1996; Solomon et al., 1999; Chen and Tyler, 2001, 2002, 2008). This collinear flanker effect is reduced as the orientation of the flanker deviates from that of the target (Polat and Sagi, 1993; Chen and Tyler, 2002), or as the flankers move away from the collinear axis toward the sides (Polat et al., 1997; Solomon et al., 1999; Chen and Tyler, 2008). Single cell recording also shows similar configuration effects (Polat et al., 1998; Kapadia et al., 2000). There is also anatomic evidence showing that V1 neurons send their fibers to contact V1 neurons in other hypercolumns with the same orientation preference (Bosking et al., 1997). Hence, there is convergent evidence for a collinear lateral interaction that is reflected in our configuration specific effect.

Many psychophysics studies demonstrate the collinear lateral interaction by showing that the detection threshold to the target decreases with the presence of collinear flankers (Polat and Sagi, 1993; Huang et al., 2012). That is, the effect of the flankers is to facilitate target detection. At first glance, this collinear facilitation contradicts our suppressive effect. However, it is known that collinear lateral interaction is contrast dependent. Polat et al. (1998, also see Chen et al., 2001) showed that the presence of collinear flankers not only increased the firing rate of the primary visual cortical neurons at low target contrast, but also decreased it at high contrast. At the behavioral level, indeed, the presence of collinear flankers reduces contrast detection and discrimination thresholds at low contrasts. However, it also increases the contrast discrimination threshold at high contrast, suggesting a reduction of internal response to the target by the flankers (Chen and Tyler, 2001, 2008; Wu and Chen, 2010). That is, the collinear lateral interaction is suppressive at high contrasts. Our stimuli had a contrast of 80%, well into the suppressive range reported in the previous studies (Polat et al., 1998; Chen and Tyler, 2001).

Some may argue that our collinear effect may be due to a preference for radial orientation in the visual cortical activation. That is, the BOLD activation of the visual cortex to a pattern whose orientation points to the fixation (radial) is greater than that whose orientation is orthogonal to the radial orientation (Sasaki et al., 2006; Freeman et al., 2013). In our experiment, the iso-orientation collinear flanker was a radial stimulus while the iso-orientation side flanker was not. Hence, the larger lateral effect produced by the collinear flanker might just reflect the greater cortical activation to the iso-orientation collinear flankers. However, notice that, the iso-orientation collinear flankers were not the only radial stimuli in our experiment. The orthogonal side flankers were also radial stimuli. Yet, we found no difference in the lateral effect produced by the side flankers (radial) and the collinear flankers (not radial) in the orthogonal condition. Hence, the radial bias of the cortical response cannot explain our result.

Different factors may underlie the general lateral suppression in our result. It is known that the presence of a visual stimulus not only produces an increment of BOLD activation in the corresponding retinotopic brain regions for that stimulus; there is also a sustained reduction in BOLD activation in the neighboring brain regions (Logothetis, 2002; Shmuel et al., 2002; Smith et al., 2004; Chen et al., 2005). One hypothesis is that negative BOLD activation may be of hemodynamic origin (Shulman et al., 1997; Shmuel et al., 2002). For instance, the presence of a visual stimulus could increase the activation of certain cortical regions, which in turn would lead to an increment of cerebral blood flow (CBF) to those cortical regions. This local increment in CBF could result in a redistribution of blood and thus a decrement of CBF in neighboring cortical regions. As a result, one may observe a decrement in BOLD activation in voxels corresponding to the visual fields outside the stimulus. Recent evidence, however, is against this “blood steal” theory. Shmuel et al. (2002, 2006) show that negative BOLD activation is correlated with the local field potential, suggesting a neural origin. Smith et al. (2004) found that negative BOLD activation can occur in a different hemisphere from the one with positive activation. Such extended signal reduction is unlikely to be hemodynamic in origin, given different blood vessels supplying the two hemispheres.

There is also evidence that the general lateral suppression of BOLD activation may be caused by the response of broadly tuned visual mechanisms. It is known that after staring at a gray region surrounded by a dynamic patterned background (adapter), observers perceive a twinkling aftereffect in the location of the gray region when the pattern stimulus is removed (Ramachandran and Gregory, 1991; Hardage and Tyler, 1995). That is, the aftereffect is induced in a region that had never received any stimulation during either the adapting or the test phases. Chen et al. (2005) showed that negative BOLD activation is positively correlated with the aftereffect. That is, while the BOLD activation in the stimulated brain region went up and down with the onset and offset of the visual stimulus respectively, the activation in the unstimulated region actually decreased after the stimulus onset and rebounded after the stimulus offset. Furthermore, the amplitude of the rebound in the unstimulated region increased the strength of the aftereffect. Thus, such negative BOLD activation should reflect the lateral inhibition in the visual system. Notice that the percept of the twinkle aftereffect is similar regardless of the pattern of the adapter. Hence, such lateral inhibition can be induced by a wide range of stimuli.

There were studies (Zenger-Landolt and Heeger, 2003; Tajima et al., 2010; Wade and Rowland, 2010) measuring the BOLD activation of a central grating surrounded by another grating. The common result was that the BOLD activation to the target can be suppressed by the presence of surrounding ring. For a better quantitative analysis for this surround effect, Wade and Rowland (2010) measured the BOLD activation to the target of various contrasts and found that their result can be fit with a model assuming a broadly tuned lateral interaction mechanism. These broadly tuned lateral interactions are consistent with the general lateral suppression we found in this study.

With a model based approach, Zuiderbaan et al. (2012) and Greene et al. (2014) showed that the BOLD activation in V1–3 to a visual stimulus can be best described by a model with a population receptive field (i.e., the receptive field of a unit of gray matter) with excitatory and inhibitory regions. This result may imply a lateral interaction among neural mechanisms. Notice that, their results were based on an analysis of single voxels while our result was manifested in ROIs with dozens of voxels. Given the difference in scale, it is difficult to make a direct comparison between the two sets of results. A further model that can associate the activation of a single and a group of voxels is needed before we can have a comprehensive treatment on the results from these different paradigms.

In conclusion, the presence of any flankers can produce a suppressive effect on BOLD activation to the central stimulus. Furthermore, it is the iso-orientated collinear flankers that create the greatest suppression. Thus, our results suggest two types of lateral suppression in BOLD activation: the first is a general suppression, which may relate to a neural mechanism with a broad tuning property, such as the one underlying “negative BOLD,” and the second is a spatial configuration dependent suppression which may be related to collinear flanker effect.

### Conflict of interest statement

The author declares that the research was conducted in the absence of any commercial or financial relationships that could be construed as a potential conflict of interest.
